# Supplementary material for: Extraordinarily transparent compact metallic metamaterials
Source: Nat Commun. 2019 May 9;10:2118. doi: 10.1038/s41467-019-09939-8 (PMC6509127; doi:10.1038/s41467-019-09939-8)
Supplement: Supplementary file 1 — Supplementary Information [file 41467_2019_9939_MOESM1_ESM.pdf]

Extraordinarily transparent compact metallic metamaterials—supplementary material

Palmer *et al*

(Dated: March 27, 2019)

SUPPLEMENTARY NOTE 1. EFFECTIVE INDEX FROM COMPLEX BAND STRUCTURE

A. Complex band structures

There are two approaches to calculating band structures. Most commonly, Maxwell's equations are written as an eigenvalue problem of the form [1]

$$\hat{\mathbf{\Theta}}(\mathbf{k})\mathbf{U}(\mathbf{k}) = \omega^2(\mathbf{k})\mathbf{U}(\mathbf{k}), \tag{S1}$$

where $\mathbf{U}(\mathbf{k})$ is the electric or magnetic field and $\hat{\mathbf{\Theta}}(\mathbf{k})$ is the corresponding eigenoperator. In this form, the frequency is typically considered to be a complex function of a real-valued wave vector, $\omega = \omega(\mathbf{k})$ [2]. However, when a material is dispersive the eigenoperator is itself a function of the eigenvalue, $\hat{\mathbf{\Theta}} = \hat{\mathbf{\Theta}}(\mathbf{k}, \omega)$, and the problem cannot be solved directly. In such cases it can be advantageous to instead consider the inverse problem, $\mathbf{k} = \mathbf{k}(\omega)$, where the Bloch wave vector is a complex function of real frequencies [3, 4], leading to *complex band structures*.

In this work we chose z as our direction of propagation (such that $k_x = k_y = 0$, see Supplementary Figure 1), and formulated a quadratic eigenvalue problem for the z-component of the Bloch wave vector, $k_z(\omega)$. From this we could determine the effective index of the metamaterial, $n_{\text{eff}} = k_z/k_0$, where $k_0 = \omega/c_0$ is the wavenumber in free space.

B. The quadratic eigenvalue problem

We begin by discretising Maxwell's equations on a grid, using either finite-differences or finite-elements, and noting the block tri-diagonal form of the wave matrix equation,

$$\mathbf{\Omega E} = \begin{bmatrix} \ddots & & & & & \\ \mathbf{a_{n-1}} & \mathbf{b_{n-1}} & \mathbf{c_{n-1}} & & & \\ & \mathbf{a_n} & \mathbf{b_n} & \mathbf{c_n} & & \\ & & \mathbf{a_{n+1}} & \mathbf{b_{n+1}} & \mathbf{c_{n+1}} & \\ & & & & \ddots & \end{bmatrix} \begin{bmatrix} \vdots \\ \mathbf{E_{n-1}} \\ \mathbf{E_n} \\ \mathbf{E_{n+1}} \\ \vdots \end{bmatrix} = \mathbf{0}, \tag{S2}$$

where $\mathbf{\Omega}$ is the wave matrix, $\mathbf{E_n} = \left[\mathbf{E}(x_1, y_1, z_n), \ldots, \mathbf{E}(x_{n_x}, y_{n_y}, z_n)\right]^T$ is the electric field in the nth 'slice' of the grid, and the $\mathbf{a_n}$, $\mathbf{b_n}$, $\mathbf{c_n}$ matrices couple the fields in the three adjacent slices, $\mathbf{E_{n-1}}$, $\mathbf{E_n}$, $\mathbf{E_{n+1}}$.

We may algebraically eliminate the degrees of freedom associated with a slice in a process that Rumpf et al [5] termed 'slice absorption'. This modifies the slice coupling matrices of neighbouring slices. For example, eliminating

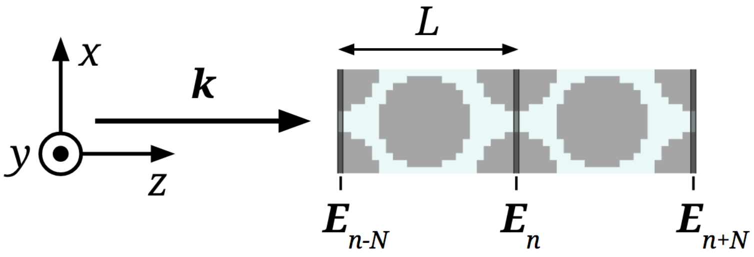

Supplementary Figure 1. The effective index is calculated using a Bloch wave ansatz. The Bloch wave vector, $\mathbf{k}$, runs parallel to the z-axis.

the nth slice produces reduced the system of equations

$$\mathbf{\Omega' E} = \begin{bmatrix} \ddots & & & \\ \mathbf{a_{n-1}} & \mathbf{b'_{n-1}} & \mathbf{c'_{n-1}} & \\ & \mathbf{a'_{n+1}} & \mathbf{b'_{n+1}} & \mathbf{c_{n+1}} \\ & & & \ddots \end{bmatrix} \begin{bmatrix} \vdots \\ \mathbf{E_{n-1}} \\ \mathbf{E_{n+1}} \\ \vdots \end{bmatrix} = \mathbf{0}, \tag{S3}$$

where

$$\mathbf{b'_{n-1}} = \mathbf{b_{n-1}} - \mathbf{c_{n-1}} \mathbf{b_n^{-1}} \mathbf{a_n} \tag{S4}$$
$$\mathbf{c'_{n-1}} = \quad - \mathbf{c_{n-1}} \mathbf{b_n^{-1}} \mathbf{c_n} \tag{S5}$$
$$\mathbf{a'_{n+1}} = \quad - \mathbf{a_{n+1}} \mathbf{b_n^{-1}} \mathbf{a}_n \tag{S6}$$
$$\mathbf{b'_{n+1}} = \mathbf{b_{n+1}} - \mathbf{a_{n+1}} \mathbf{b_n^{-1}} \mathbf{c_n}. \tag{S7}$$

In order to calculate the effective index in a z-periodic system we first absorb all but one of the slices within each unit cell,

$$\begin{bmatrix} \ddots & & & & \\ \mathbf{a} & \mathbf{b} & \mathbf{c} & & \\ & \mathbf{a} & \mathbf{b} & \mathbf{c} & \\ & & \mathbf{a} & \mathbf{b} & \mathbf{c} \\ & & & & \ddots \end{bmatrix} \begin{bmatrix} \vdots \\ \mathbf{E_{n-N}} \\ \mathbf{E_n} \\ \mathbf{E_{n+N}} \\ \vdots \end{bmatrix} = \mathbf{0}, \tag{S8}$$

as illustrated in Supplementary Figure 1. Note that the modified coupling matrices are periodic, $\mathbf{a} = \mathbf{a_n} = \mathbf{a_{n+N}}$, etc, and fully dense.

The quadratic eigenvalue problem is obtained from the Bloch wave ansatz $\mathbf{E}(\mathbf{r}, \omega) = \mathbf{u}(\mathbf{r}) e^{ik_z z - i\omega t}$, where $\mathbf{u}(\mathbf{r}+\mathbf{R}) = \mathbf{u}(\mathbf{r})$ for any lattice vector $\mathbf{R}$,

$$(\mathbf{a} e^{-ik_z L_z} + \mathbf{b} + \mathbf{c} e^{+ik_z L_z}) \mathbf{u} = \mathbf{0}. \tag{S9}$$

C. Solution of the quadratic eigenvalue problem

In general, quadratic eigenvalue problems may be solved via linearisation [6]. For example, the quadratic eigenvalue problem $(\lambda^2 \mathbf{M} + \lambda \mathbf{C} + \mathbf{K})\mathbf{x} = \mathbf{0}$ can be linearised by the substitution $\mathbf{u} = \lambda \mathbf{x}$,

$$\begin{bmatrix} \mathbf{0} & \mathbf{I} \\ -\mathbf{K} & -\mathbf{C} \end{bmatrix} \begin{bmatrix} \mathbf{x} \\ \mathbf{u} \end{bmatrix} - \lambda \begin{bmatrix} \mathbf{I} & \mathbf{0} \\ \mathbf{0} & \mathbf{M} \end{bmatrix} \begin{bmatrix} \mathbf{x} \\ \mathbf{u} \end{bmatrix} = \begin{bmatrix} \mathbf{0} \\ \mathbf{0} \end{bmatrix}. \tag{S10}$$

Note that the linearised eigenvalue problem has twice as many degrees of freedom as the quadratic eigenvalue problem.

However, when the system is symmetric under z-inversion or t-reversal, then Supplementary Equation (S9) is invariant under $k_z \leftrightarrow -k_z$,

$$(\mathbf{a} e^{ik_z L_z} + \mathbf{b} + \mathbf{c} e^{-ik_z L_z}) \mathbf{u} = \mathbf{0}. \tag{S11}$$

For such systems we can solve directly for $\cos k_z L_z$ without increasing the number of degrees of freedom by summing Supplementary Equations (S9) and (S11),

$$[(\mathbf{a} + \mathbf{c}) \cos(k_z L_z) + \mathbf{b}] \, \mathbf{u} = \mathbf{0}. \tag{S12}$$

Note that as $\cos(k_z L_z)$ is an even function of k_z, and Supplementary Equation (S12) is therefore invariant under $k_z \leftrightarrow -k_z$ in accordance with the symmetry of the system.

Regardless of the method of solution, the real part of $k_z L_z$ is ambiguous. However, for frequencies low enough that the effective wavelength is much larger than the unit cell, $k_z L_z \ll \pi$, we can assume that the mode lies in the first Brillouin zone, $|k_z| < \pi / L_z$.

Veres et al [3] used a process of algebraic elimination similar to the slice absorption method to calculate complex band structures of phononic crystals. However, they retained the degrees of freedom of the entire cell perimeter, not just those of one slice. For an approximately square unit cell, our approach reduces the degrees of freedom by a factor of four. Combined with Supplementary Equation (S12) for systems with z-inversion or t-reversal symmetry, the degrees of freedom are reduced by a total factor of eight compared to the approach of Veres et al, such that the memory to store the matrix eigenvalue problem is reduced by a factor of 64.

SUPPLEMENTARY NOTE 2. WEAK MAGNETIC RESPONSES

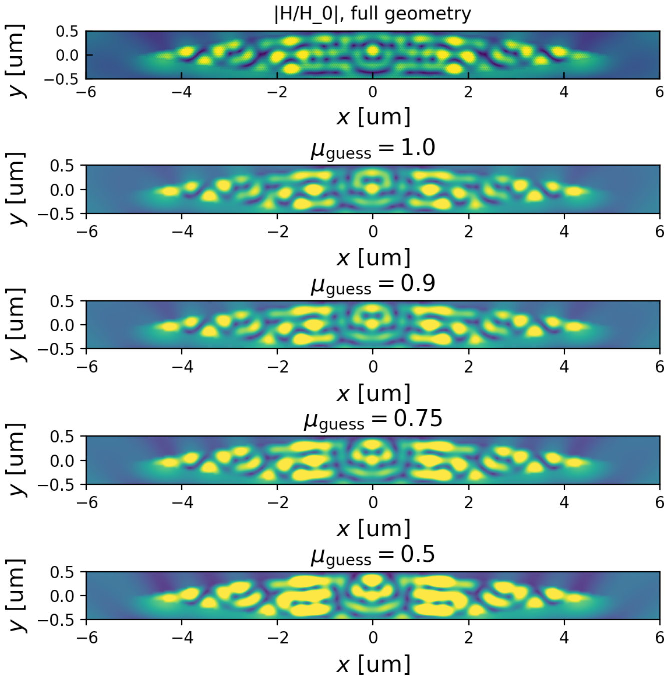

Supplementary Figure 2. The magnetic near-field of the lens. The solution from figure 3d is compared to the magnetic near-fields of lenses with the same effective refractive index, n_{eff}, but different effective magnetic responses. Qualitatively, the behaviour of the full geometry is best approximated by a weak effective magnetic response, $\mu_{\mathrm{eff}} \gtrsim 0.9$

.

In Figures 2a, 2b, 3e, and 5c of the main text, the systems are assumed to have a negligible magnetic response, $\mu_{\mathrm{eff}} \approx 1$, such that the effective permittivity can be approximated as $\epsilon_{\mathrm{eff}} \approx \sqrt{n_{\mathrm{eff}}}$. It can be seen qualitatively in Supplementary Figure 2 that this assumption is acceptable, as agreement between the full geometry and homogenised simulation is greatest for $\mu_{\mathrm{eff}} \approx 1$. These simulations were for our 'worst case' scenario of a relatively short wavelength ($\lambda_0 = 2\,\mu\mathrm{m}$) and relatively large particles ($d = 38\,\mathrm{nm}$, G=2 nm).

SUPPLEMENTARY NOTE 3. ORIGINS OF THE DISPERSION AT NEAR-TO-MID INFRARED WAVELENGTHS

In Figure 4a of the main text (shown again here in Supplementary Figure 3c for a smaller range of wavelengths) the effective index of the titanium nanoparticle array peaks at $\lambda_0 \approx 5\,\mu\mathrm{m}$, while the effective index dips for the other metals. Here we discuss the origins of these peaks and dips.

We begin by considering the Maxwell-Garnett mixing formula in Supplementary Figure 3a. Overall, the effective index is lower because the particle-particle interactions are neglected. Although we still observe the peak in the titanium effective index with the MG formula, we do not see the dips in effective index for the other metals, suggesting that the peak and the dips have different origins.

In the case of titanium, there is a peak in the effective index because titanium is a poor metal (ϵ is not very large or negative) around $\lambda_0 = 5\,\mu\mathrm{m}$. As shown in Equation (4) of the main text, the MG mixing formula only tends to a constant for materials with large ϵ.

The dips in the effective index of the other metals, which are not replicated by the MG mixing formula, occur because the quasi-static approximation breaks down at smaller wavelengths as the skin depth of the metals shrinks. This drop is consistent with the behaviour seen in Figure 4b of the main text. The quasi-static approximation can be restored and the size of the dip reduced by making the particles smaller, as in Supplementary Figure 3b.

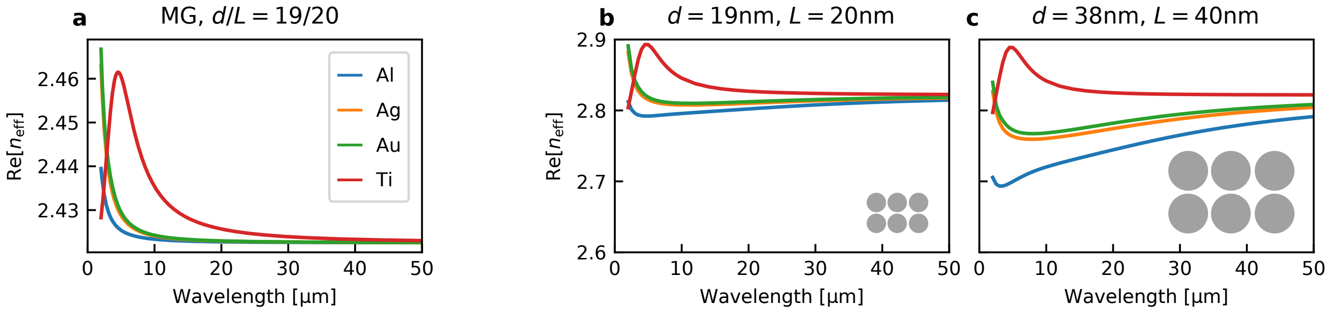

Supplementary Figure 3. Dispersion in the near-to-mid infrared spectrum. (a) Effective index of square arrays of metallic cylinders, calculated over a range of wavelengths using the Maxwell Garnett mixing formula. (b) When the particles are small compared to the skin depth, the full wave simulations of the effective index are qualitatively similar to those of MG, although the effective index is raised by the particle-particle interactions. (c) For larger particles, as in the main text, the effective index drops at smaller wavelengths where the skin depth is large compared to the particle diameter and the quasi-static approximation breaks down.

SUPPLEMENTARY NOTE 4. LOSSES IN THE TELECOM AND VISIBLE WAVELENGTHS

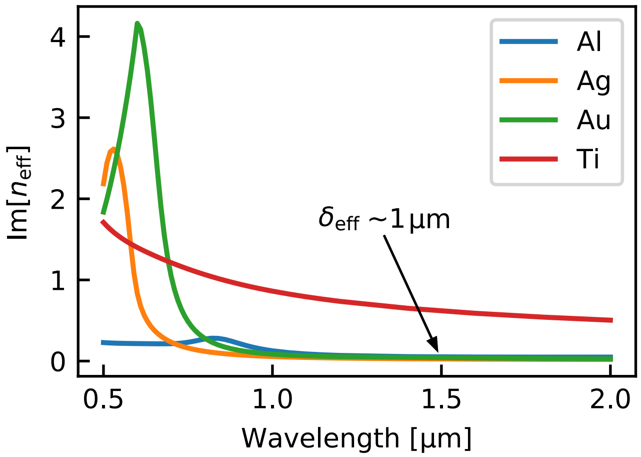

Supplementary Figure 4. Losses in the telecom and visible wavelengths. Losses are given by the imaginary part of the effective index, for metallic nanocylinders on a triangular lattice. The particles have diameter $d = 38\,\text{nm}$ and surface-to-surface separation $G = 2\,\text{nm}$.

As shown in Supplementary Figure 4, the imaginary part of the effective index remains relatively low into the telecoms wavelengths for all metals except titanium. At $\lambda_0 = 1.5\,\mu\text{m}$, the effective penetration length of the arrays, $\delta_{\text{eff}} = \lambda_0/4\pi\text{Im}[n_{\text{eff}}]$, is on the order of a micron. Although this is shorter at infrared wavelengths where the skin depth can be on the order of centimeters (see for example Figure 2b of the main text), it is still sufficiently transparent for sub-micrometer devices. However, by the time we reach the visible wavelengths, the quasi-static dipole approximation is broken for all metallic particle of realistic diameters, and the losses are very high.

One may ask, if titanium has the longest skin depth of the four metals, and we establish a general trend between large skin depths and higher transparency, why does titanium produce the lossiest metamaterial at $\lambda_0 = 2$? It is because although it is well described by quasi-static dipoles and the Maxwell Garnett mixing formula (Equation 4 of main text),

$$\epsilon_{\text{MG}}^{\text{cyl}} \approx \frac{1+f}{1-f} - \frac{4f}{(1-f)^2}\frac{1}{\epsilon} + \mathcal{O}\left(\frac{1}{\epsilon^2}\right). \tag{S13}$$

the permittivity of titanium at $\lambda_0 = 2\,\mu\text{m}$ is relatively small, $|\epsilon| \approx 40$, and the lossy $1/\epsilon$ term is relatively large, compared to the other metals. In essence, titanium is not a good metal at these wavelengths.

SUPPLEMENTARY NOTE 5. ACCURACY OF THE MAXWELL-GARNETT MIXING FORMULA FOR SQUARE AND TRIANGULAR LATTICES

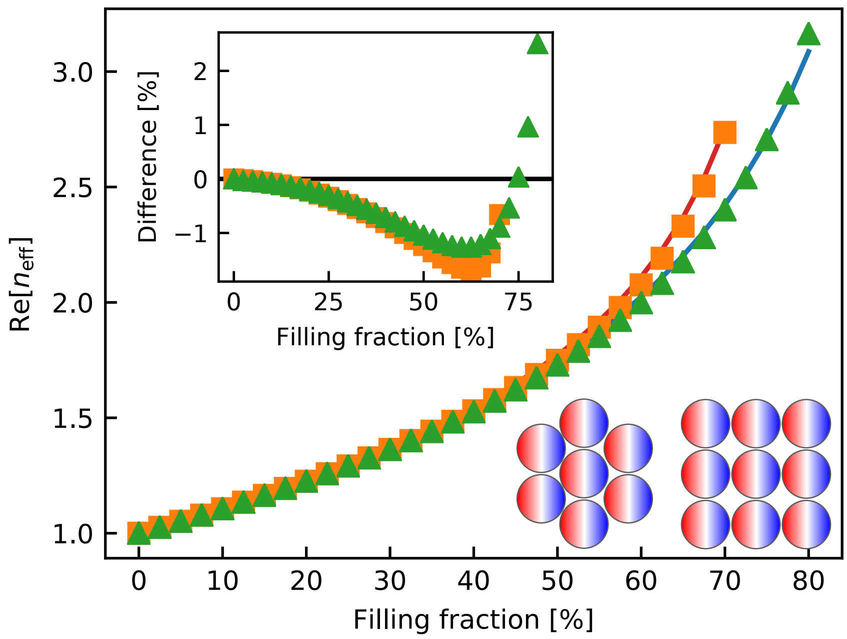

Supplementary Figure 5. Interparticle interactions depend on the arrangement of the nanocylinders. The effective indices of metallic cylinder arrays are compared to the Maxwell Garnett mixing formula (blue curve) and the Rayleigh formula (orange curve) for both square and triangular lattices (bottom right insets), with a fixed lattice parameter $L = 40\,\text{nm}$. The filling fraction is varied, up to a minimum surface-to-surface separation of 2 nm. The cylinders are made of gold and the wavelength is $\lambda_0 = 2\,\mu\text{m}$.

Supplementary Figure 5 compares the accuracy of the Maxwell Garnett mixing formula when describing square and triangular lattices of metallic nanocylinders. As expected, a higher effective index can be achieved using a triangular lattice as the maximum filling fraction is larger than that of the square lattice. However, as shown in the inset, the dipoles in square arrays are more directly aligned than those in the triangular lattice. This leads to stronger dipole-dipole interactions between the cylinders in the square lattice. The Maxwell Garnett mixing formula neglects interactions between the inclusions, which act to increase the effective polarisability of the array, and consequently underestimates the effective index of the square lattice.
The Rayleigh formula is an extension of the MG mixing formula that takes into account particle-particle interactions, up to a certian order. For cylinders in a square lattice, the Rayleigh formula is [7]

$$\epsilon_{\text{eff}} = 1 + \frac{2f}{\frac{\epsilon_r+1}{\epsilon_r-1} - f - \frac{\epsilon_r-1}{\epsilon_r+1}\left(0.3058f^4 + 0.0134f^8\right)}. \tag{S14}$$

In principle, a similar formula could be found for cylinders on a triangular lattice, but as we have seen in Supplementary Figure 5, MG is sufficiently accurate for the purposes of this work, especially for cylinders on a triangular lattice.

SUPPLEMENTARY NOTE 6. MAXWELL-GARNETT MIXING FORMULA FOR ELLIPTICAL CYLINDERS

To obtain the Maxwell-Garnett mixing formula for elliptical cylinders, we first need to derive the electrostatic polarisability of an elliptical cylinder, α_{qs}. We can then find the effective permittivity from the effective polarisability, $\alpha_{\text{eff}} = f\alpha_{\text{qs}}$.

A. Equation of an ellipse in elliptic cylindrical coordinates

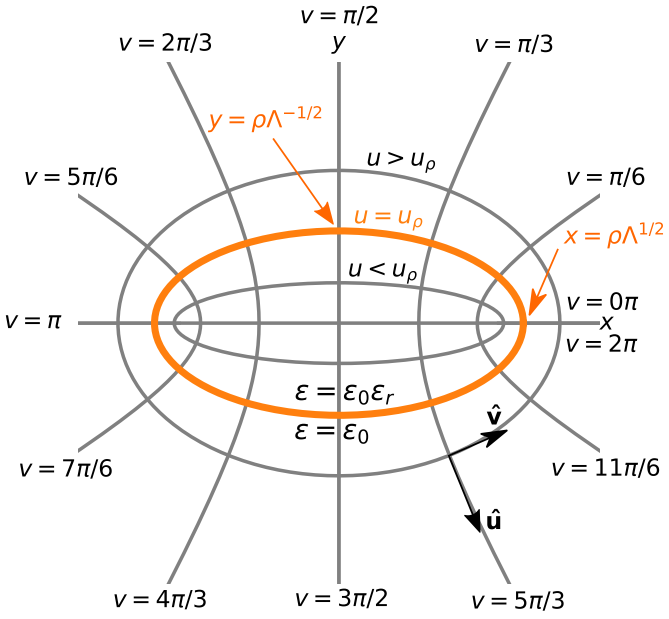

Supplementary Figure 6. Elliptic cylindrical coordinates, with lines of constant u and v. Note that lines of constant u form elliptic cylinders, and the aspect of each cylinder is controlled by the magnitude of u.

Elliptic cylindrical coordinates are defined as [8]

$$x = a \cosh u \cos v \tag{S15a}$$

$$y = a \sinh u \sin v \tag{S15b}$$

where $u = [0, \infty)$, $v = [0, 2\pi)$. Note that v is an angular coordinate that runs parallel to the surface of the ellipse, whereas u is a radial coordinate that runs normal to the surface of the ellipse, as shown in 6.

The equation of an elliptic cylinder with aspect ratio $\Lambda = L_x/L_y$, defined in Cartesian coordinates as

$$\frac{x^2}{\Lambda} + \frac{y^2}{1/\Lambda} = \rho^2. \tag{S16}$$

may be written in elliptic cylindrical coordinates as

$$u = u_\rho = \tanh^{-1}(1/\Lambda), \tag{S17}$$

$$a = \sqrt{\Lambda - 1/\Lambda}\rho. \tag{S18}$$

Laplace's equation

Assuming $\partial_z \phi = 0$, the form of Laplace's equation is unchanged when we transform to elliptic cylindrical coordinates,

$$\left(\frac{\partial^2}{\partial u^2} + \frac{\partial^2}{\partial v^2} \right) \phi(u, v) = 0. \tag{S19}$$

By separation of variables we can write

$$\phi(u, v) = U(u)V(v), \tag{S20}$$

with

$$\frac{\mathrm{d}^2U}{\mathrm{d}u^2} = n^2U, \tag{S21}$$

$$\frac{\mathrm{d}^2V}{\mathrm{d}v^2} = -n^2V, \tag{S22}$$

for constant of separation n. This has solutions $\phi_n = U_nV_n$, where

$$V_n = a_n\cos(nv) + b_n\sin(nv), \tag{S23}$$

$$U_n = c_n\cosh(nu) + d_n\sinh(nu). \tag{S24}$$

Angular periodicity

We know that we have the angular periodicity $V_n(v) = V_n(v + 2\pi)$, so $\cos(nv) = \cos(nv + 2\pi n)$, such that n is an integer.

Symmetry about x-axis

The geometry and the applied field are both symmetric about the x-axis. Therefore we know that V_n should be even about $v = 0$, such that $b_n = 0$,

$$V_n = a_n\cos(nv)$$

Potential inside the particle

Inside the particle, we use the ansatz that the field will be uniform inside the particle in the electrostatic limit,

$$\phi_{\text{in}} = -AE_0x. \tag{S25}$$

In order to calculate the electrostatic polarisability of the particle, we must first solve for A.

Potential outside the particle

For an applied field $\mathbf{E} = E_0\hat{\mathbf{x}}$, the electric potential far from the scatterer must tend to $\phi_{\text{out}}(u\to\infty) = -E_0x$. This is satisfied by

$$\phi_{\text{out}} = -E_0x + \sum_{n=1}^{\infty} U_nV_n, \tag{S26}$$

provided that we choose $d_n = -c_n$, such that

$$U_n = c_n\cosh(nu) - c_n\sinh(nu), \tag{S27}$$

$$= c_ne^{-nu}, \tag{S28}$$

and $U_nV_n \to 0$ for $u \to \infty$ for all n.

Therefore the potential outside the particle is of the form

$$\phi_{\text{out}} = -E_0x + \sum_{n=1}^{\infty} B_n\cos(nv)e^{-nu} \tag{S29}$$

where $B_n = a_nc_n$.

Maxwell's interface conditions

We may find A from Maxwell's interface conditions for the electric field. The tangential component of the E-field is continuous at the surface,

$$\left(\frac{\partial \phi_{\text{in}}}{\partial v}\right)_{u=u_\rho} = \left(\frac{\partial \phi_{\text{out}}}{\partial v}\right)_{u=u_\rho} \tag{S30}$$

such that

$$\left(\frac{\partial \phi_{\text{in}}}{\partial v}\right)_{u=u_\rho} = (a \cosh u_\rho \sin v) A E_0, \tag{S31}$$

$$\left(\frac{\partial \phi_{\text{out}}}{\partial v}\right)_{u=u_\rho} = (a \cosh u_\rho \sin v) E_0 - \sum_{n=1}^{\infty} B_n n \sin(nv) e^{-n u_\rho}, \tag{S32}$$

where we have used $\partial_v x = -a \cosh u \sin v$. Therefore, by equating coefficients of $\sin(nv)$, we see that $B_{n\neq 1} = 0$, and

$$e^{-u_\rho} B_1 = (1 - A) a \cosh u_\rho \cdot E_0. \tag{S33}$$

Similarly, the normal component of the D-field is continuous at the surface,

$$\epsilon_r \left(\frac{\partial \phi_{\text{in}}}{\partial u}\right)_{u=u_\rho} = \left(\frac{\partial \phi_{\text{out}}}{\partial u}\right)_{u=u_\rho}, \tag{S34}$$

such that

$$e^{-u_\rho} B_1 = (\epsilon_r A - 1) a \sinh u_\rho \cdot E_0, \tag{S35}$$

where we have used $\partial_u x = a \sinh u \cos v$. By equating Supplementary Equations (S33) and (S35), and recalling that $\tanh u_\rho = 1/\Lambda$, we find that

$$A = \frac{1 + \Lambda}{\epsilon_r + \Lambda}. \tag{S36}$$

Electrostatic polarisability

Now we know the internal electric potential, we can find the electrostatic polarisability,

$$\mathbf{P} = (\epsilon - 1)\mathbf{E}_{\text{in}} = (\epsilon - 1) A \mathbf{E}_0 = \alpha_{\text{qs}} \mathbf{E}_0, \tag{S37}$$

such that

$$\alpha_{\text{qs}} = \frac{(\epsilon - 1)(1 + \Lambda)}{\epsilon_r + \Lambda} \cdot V. \tag{S38}$$

Maxwell Garnett Mixing Formula

Now we search for a homogeneous medium with an effective polarisability, $\alpha_{\text{eff}} = f \alpha_{\text{qs}}$, that is equivalent to the polarisability of the (non-interacting) particles,

$$\frac{(\epsilon_{\text{eff}} - 1)(1 + \Lambda)}{\epsilon_{\text{eff}} + \Lambda} = f \frac{(\epsilon - 1)(1 + \Lambda)}{\epsilon_r + \Lambda}, \tag{S39}$$

leading to

$$\epsilon_{\text{eff}} = \frac{\epsilon_r + \Lambda + \Lambda f(\epsilon_r - 1)}{\epsilon_r + \Lambda - f(\epsilon_r - 1)}. \tag{S40}$$

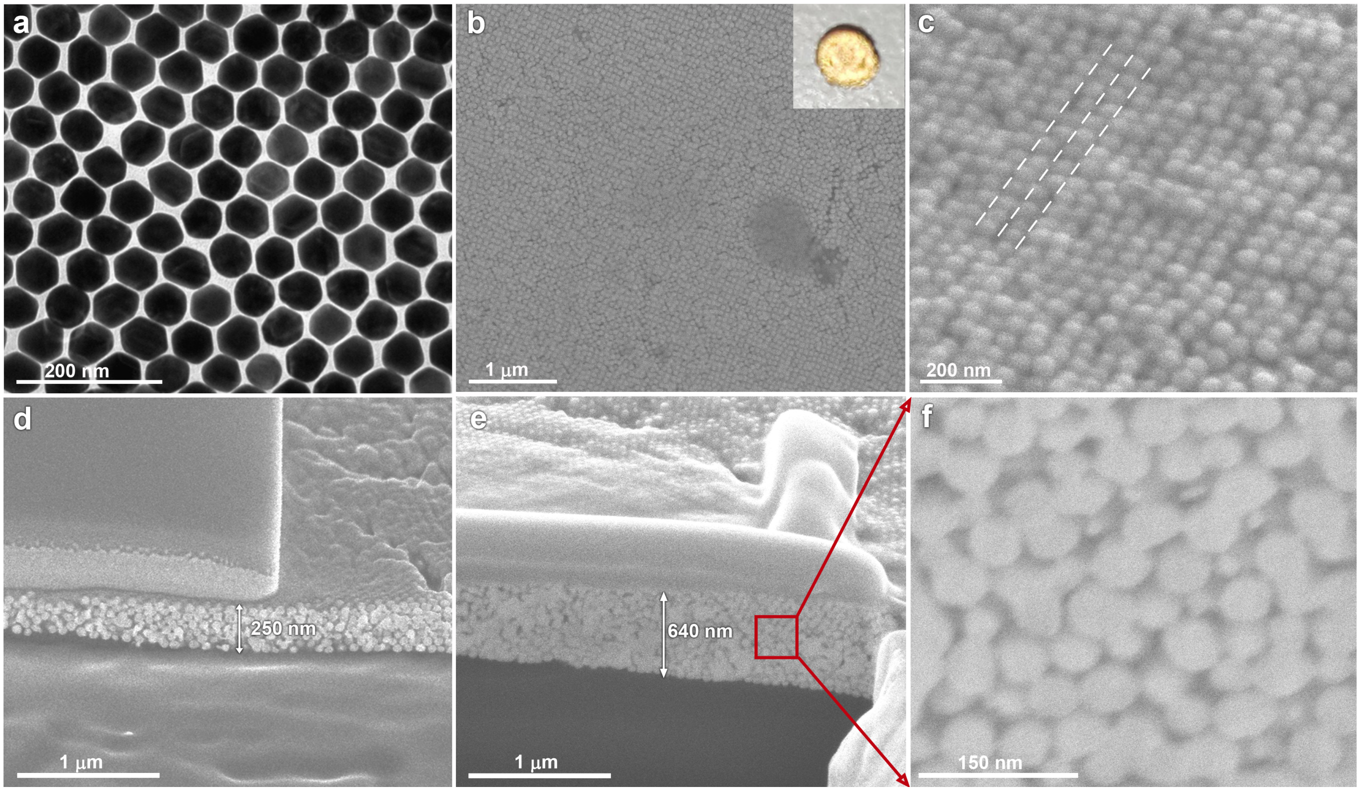

Supplementary Figure 7. (a-f) Microscopy images of gold colloidal supercrystals deposited on germanium, and a sample as seen without magnification (b, inset).

SUPPLEMENTARY NOTE 7. EXPERIMENT

A. Synthesis of spherical gold nanoparticles

CTAB-stabilized spherical gold nanoparticles of approx. 60 nm in diameter were produced according to a previously reported seed mediated procedure [9]. Briefly, small Au seed particles were prepared by adding sodium citrate and $HAuCl_4$ to 20 mL of water to achieve a final concentration of 2.5×10^{-4} M for both reactants. Subsequently, $NaBH_4$ (600 $\sim$ 5 μL, 0.1 M) was quickly injected while the solution was being energetically stirred (1200 rpm). Stirring was continued for 1 h under an open atmosphere to allow the decomposition of $NaBH_4$ and, thus, avoid overpressure. Next, a growth solution was prepared by dissolving CTAB in Milli-Q water (f.c. 0.1 M, 1000 mL) followed by the addition of $HAuCl_4$ (0.112 M, 4216 $\sim$ 5 μL), NaI (0.1 mM, 138 $\sim$ 5 μL) and ascorbic acid (0.1 M, 7350 $\sim$ 5 μL). After each addition, the reaction vessel was vigorously shaken. Immediately after, 3 mL of the as-prepared seeds were carefully added into the foam of the growth solution, the mixture was vigorously shaken, and the bottles were left undisturbed at 32° C for 48 h. Subsequently, a sediment in the bottom of the flask was observed. In order to maximize nanoparticle monodispersity, the supernatant was carefully collected, and the precipitate was discarded. Afterwards, the gold NPs were concentrated to 250 mL by centrifuging at 7500 rpm for 10 minutes and redispersing in a 0.1 M CTAB solution.

B. Colloidal crystals formation

Just before the crystal assembly, the CTAB-stabilized spherical gold nanoparticles where concentrated and cleaned to achieve concentrations of 20 mg mL^{-1} and 1 mM of Au NPs and CTAB. To do this, 5 mL of the Au NPs were centrifuged three times at 7000 rpm for 10 min. After each centrifugation step, the nanoparticles were progressively concentrated (at 2 mL, 0.5 mL, and 0.1 mL) meanwhile cleaning the CTAB excess by dispersing them using a CTAB solution 1 mM. The production of the NPs crystals was performed by a dry confinement process [9]. This was achieved by cutting a 1 cm$\times$1 cm square of PDMS with a hole in the middle (3 mm diameter). Next, the PDMS was placed

on the Ge substrate (1 mm in thickness) and the hole was filled with 10 $\sim$ 5 µL of the concentrated NP solution (Au^0 = 20 mg mL^{-1}, CTAB= 1 mM). The whole setup was placed in a humidity chamber to avoid evaporation of the solvent, enabling the particles to sediment. After the particles have sediment (approx. 48 h), the chamber was open to evaporate the water.

C. Characterization

Optical characterization was carried out by UV/Vis/NIR spectroscopy with a Cary 5000 spectrophotometer. Normal and cross-sectional SEM images of the nanoparticle supercrystals were acquired with a FIB workstation with a FEI Helios 400 NanoLab dual-beam microscope. A dedicated backscattered electron detector (Autrata YAG detector) was used to enhance the signal. Note that the irregularities on the gold nanoparticles observed in the cross-sectional image are due to the redeposition of the gold vapor generated during the exposition of the supercrystal to the focused ion beam.

D. Materials

Gold(III) chloride trihydrate (99.9%, $HAuCl_4{\cdot}3H_2O$), trisodium citrate dihydrated (99.5%, $C_6H_5Na_3O_7{\cdot}2H_2O$), and ascorbic acid (99.9%) were purchased from Sigma-Aldrich (Germany). N-Cetyl-N,N,N-trimethylammonium bromide (99.72-%, CTAB) was acquired from Merck. Poly(dimethylsiloxane) (PDMS) silicon elastomer (Sylgard 184 base and curing agent) was obtained from Dow Corning. All reactants were used without further purification. Milli-Q water (18 MΩ cm^{-1}) was used in all aqueous solutions, and all the glassware were cleaned with aqua regia before use.

SUPPLEMENTARY REFERENCES

[1] Gangaraj, S. A. H., Silveirinha, M. G. & Hanson, G. W. Berry phase, Berry Connection, and Chern Number for a Continuum Bianisotropic Material from a Classical Electromagnetics Perspective **2**, 3–17 (2016). URL `http://dx.doi.org/10.1109/JMMCT.2017.2654962`. 1611.01670.

[2] Johnson, S. & Joannopoulos, J. Block-iterative frequency-domain methods for Maxwell's equations in a planewave basis. *Optics Express* **8**, 173 (2001). URL `https://www.osapublishing.org/oe/abstract.cfm?uri=oe-8-3-173`.

[3] Veres, I. A., Berer, T. & Matsuda, O. Complex band structures of two dimensional phononic crystals: Analysis by the finite element method. *Journal of Applied Physics* **114** (2013).

[4] Reuter, M. G. A unified perspective of complex band structure: interpretations, formulations, and applications. *Journal of Physics: Condensed Matter* **29**, 053001 (2016).

[5] Rumpf, R. C., Tal, A. & Kuebler, S. M. Rigorous electromagnetic analysis of volumetrically complex media using the slice absorption method. *JOSA A* **24**, 3123–3134 (2007).

[6] Tisseur, F. & Meerbergen, K. The quadratic eigenvalue problem. *SIAM review* **43**, 235–286 (2001).

[7] Wallén, H., Kettunen, H. & Sihvola, A. Mixing formulas and plasmonic composites. In *Metamaterials and plasmonics: fundamentals, modelling, applications*, 91–102 (Springer, 2009).

[8] Weisstein, E. W. Elliptic cylindrical coordinates (2003).

[9] Tebbe, M. *et al.* Fabrication and optical enhancing properties of discrete supercrystals. *Nanoscale* **8**, 12702–12709 (2016).
